# Prevalence of white matter hyperintensities and radiological cerebral small vessel disease: an insight from routinely collected data

**DOI:** 10.1186/s12883-025-04557-y

**Published:** 2025-12-19

**Authors:** Mark P. Maskery, Nicola Rennie, Sachin Mathur, Jo Knight, Hedley C. A. Emsley

**Affiliations:** 1 Lancaster Medical School, Lancaster University, Lancaster, UK; 2 Department of Neurology, Lancashire Teaching Hospitals NHS Foundation Trust, Sharoe Green Lane, Fulwood, Preston, PR2 9HT UK; 3 Department of Neuroradiology, Lancashire Teaching Hospitals NHS Foundation Trust, Preston, UK

**Keywords:** Magnetic resonance imaging, Stroke, Small vessel disease

## Abstract

**Background:**

Approximately 900,000 MRI brain scans are performed annually in the United Kingdom alone, with incidental findings frequently encountered.

One of the most prevalent findings is white matter hyperintensities (WMHs). WMHs often indicate cerebral small vessel disease (cSVD) but can also be associated with migraine and demyelination. Prospective population studies have already confirmed a high prevalence of WMHs in elderly patients. In younger patients, or when the radiological burden is low, WMHs are commonly considered non-specific. Routinely collected data represents a valuable resource to facilitate further study. We aimed to describe the prevalence of WMHs in a direct to scan referral population and to understand associations with age, demographics, performance status and referral criteria.

**Methods:**

We performed a service evaluation of our local two-week wait suspected central nervous system cancer pathway to understand the association between age, demographics, performance status, referral criteria, imaging outcomes and both the prevalence and radiological characteristics of WMHs. Analysis was performed using R version 4.1.3.

**Results:**

We identified 1033 patients, referred over a 30-month period. Mean patient age was 51.3±18.3 years with 65% females. As expected, WMHs were present on 89.7% of scans in patients aged over 80, with 98.1% of these consistent with cSVD upon review by an experienced neuroradiologist. We show an important association between WMHs deemed representative of cSVD and both performance status and levels of deprivation.

However, WMHs were also present in approximately 1 in 5 patients under 50 years old and were typically deemed non-specific. Our analysis showed prevalence of WMH, its radiological burden and likelihood of WMHs being attributed to cSVD all increased with age. It is therefore feasible to consider that these changes may represent early cSVD.

**Conclusions:**

We demonstrate a prevalence of radiological cSVD comparable to the wider literature in elderly patients whilst highlighting the potential underestimation of cSVD in the younger population, in whom further study of WMHs is required.

There is potential for routinely collected data to define the prevalence and characteristics of radiological cSVD more accurately whilst facilitating further research.

**Supplementary Information:**

The online version contains supplementary material available at 10.1186/s12883-025-04557-y.

## Introduction

Cerebral small vessel disease (cSVD) represents a highly prevalent spectrum of disorders resulting in significant healthcare burden worldwide [1–4]. 

Often detected incidentally, radiological features of cSVD include white matter hyperintensities (WMHs) of presumed vascular origin, lacunes, prominent vascular spaces, cerebral microbleeds and atrophy [5]. WMHs can also be associated with migraine, demyelination and traumatic brain injury [6–8]. Assessment of the spatial distribution and morphology of WMHs can indicate their radiological aetiology, but when the burden of lesions is low they are often deemed non-specific [9]. 

Clinically cSVD contributes to 20–25% of stroke and 45% of dementia, however a frequently overlooked prodrome of vascular parkinsonism, gait disturbance, falls, apathy, depression, incontinence and/or an emergent dysexecutive cognitive profile may be apparent upon clinical assessment [10–12]. cSVD is also implicated in the development of later life epilepsy [13]. When, or indeed whether, these clinical phenotypes materialise may be associated with individual physiological reserve, compensatory mechanisms or may represent the heterogenous underlying pathophysiology [14, 15]. Rather than an ubiquitous component of the normal aging process, it is clear that radiological evidence of cSVD bestows adverse physical and prognostic implications [16–18]. 

The management of incidentally discovered cSVD, evident on imaging performed for alternate reasons, is largely directed by the recent ESO guideline [19]. The mainstay of therapy comprises simple holistic measures such as lifestyle modification and hypertension management [14]. Several studies support active surveillance and management of hypertension in cSVD, with a target systolic blood pressure of < 130mmHg associated with reduced WMH progression [20–22]. Otherwise, there is no specific disease modifying therapy for cSVD and antiplatelet therapy is not recommended unless indicated for other co-existent factors such as previous stroke or transient ischaemic attack, nor do recommendations directly address covert cSVD in younger populations [19, 23]. 

Addressing this significant public health concern is vital, particularly with an ageing population. Prospective population studies have already demonstrated the high prevalence of WMHs in this elderly cohort, but with increasing availability of electronic patient records and approximately 900,000 MRI brain scans performed in the United Kingdom annually, there is considerable scope for routinely collected data to improve our understanding of cSVD prevalence, demographics and risk factors throughout the wider population [24–26]. 

The aim of the study was to describe the prevalence and radiological characteristics of WMHs, with a focus on cSVD, amongst a population with suspected brain cancer referred directly for brain scans. We also aimed to understand the associations with age, demographics, performance status and referral criteria.

## Methods

We identified our local two week wait (2WW) referral pathway for suspected central nervous system (CNS) cancer at Lancashire Teaching Hospitals NHS Foundation Trust, a regional neurosciences centre in the North West of England, as a potential data source for further analysis. Patients meeting certain referral criteria, including recent onset headache, rapidly progressive or subacute focal neurological deficit and/or other symptoms attributable to the central nervous system are routinely invited for an expedited direct-to-scan MRI brain appointment. Patients undergo a 1.5T MRI brain including T1, T2, diffusion weighted imaging (DWI) and susceptibility weighted imaging (SWI) followed by discussion in a mini-multidisciplinary team (MDT) meeting attended by a consultant neurologist with a specialist interest in cerebrovascular disease and a consultant neuroradiologist. Considering the clinical information available on the structured referral proforma in the context of the radiological findings there is direct feedback to the referring clinician to guide ongoing care. The purpose of this 2WW CNS MDT is to expeditiously exclude brain malignancy, present in only 3% of cases upon a previous study of this referral stream, though further assessment by neurology can be initiated should this still be required [27]. We are therefore unable to obtain the final neurological diagnosis via this source of routinely collected data.

We retrospectively reviewed patient demographics, structured referral documentation and imaging outcomes between June 2020 and January 2023. This included patient’s age, sex, World Health Organisation (WHO) performance status classification (see Table 1), 2WW referral criteria, imaging modality and findings. For each scan, the attending consultant neurologist and neuroradiologist routinely evaluated for the presence of WMHs, their suspected radiological aetiology and included a subjective measure of radiological burden. WMH aetiology (non-specific/equivocal, vascular or demyelinating) was based upon morphology, topographical distribution and an overall review of imaging for other features in keeping with cSVD as per the Standards for Reporting Vascular Changes on Neuroimaging (STRIVE) definitions [5]. WMH burden was measured between 0 and 5 on a visual scale (0 – Nil present, 1 – Mild/few, 2 – Mild to moderate, 3 – Moderate, 4 – Moderate to severe, 5 – Severe/extensive).

**Table 1:** World Health Organisation (WHO) performance status classification

0 - Able to carry out all normal activity without restriction 1 - Restricted in strenuous activity but ambulatory and able to carry out light work 2 - Ambulatory and capable of all self-care but unable to carry out any work activities; up and about more than 50% of waking hours 3 - Symptomatic and in a chair or in bed for greater than 50% of the day but not bedridden 4 - Completely disabled; cannot carry out any self-care; totally confined to bed or chair	

For our initial analysis, all WMHs were included regardless of their suspected aetiology to assess their overall prevalence. WMHs were subsequently delineated into groups deemed radiologically to be ‘cSVD’ (WMHs of presumed vascular aetiology, grade 1–5) vs. ‘no cSVD’ (no WMHs present upon imaging or WMHs present were deemed non-specific/equivocal or demyelinating).

In England and Wales, postcodes are grouped into output areas, which are then combined into lower-layer super output areas (LSOAs). LSOAs consist homogenously of approximately 1500 people, for which a selection of small-area reporting data (i.e., Census-based) is available. Postcode data was therefore cross-referenced with LSOAs to obtain 2019 UK government Open Data deciles for measures including the overall Index of Multiple Deprivation (IMD), Education, Skills and Training Deprivation (EST) and Health Deprivation and Disability (HDD) [28]. 

Counts and percentages were used to explore demographics and report prevalence. Chi-squared tests were used to confirm apparent lack of association of variables between groups. For the trends that appear positive we have not performed statistical tests because these associations are not adjusted for other variables and further multivariable analysis is performed in the cSVD cohort. Two separate logistic regression models were fitted to assess the relationship between cSVD and other variables; in the first patient demographics were explored with a multivariable logistic regression and the second referral criteria/symptoms were explored with a conditional logistic regression, conditioned on age. cSVD is known to be associated with age but to explore the strength of this in our data we undertook a sensitivity analysis by randomly flipping 10% of cSVD labels and fitting models with age, age remained extremely significant in all cases hence its inclusion in both models. For the first model demographic variables that were plausible risk factors were selected from the demographic variables available. We have not included correction for multiple testing as this was an exploratory study that has focused on the presentation of descriptive statistics.

This study was based entirely on retrospective analysis of routinely collected data and was deemed to represent a service evaluation registered at Lancashire Teaching Hospitals NHS Foundation Trust. Ethics approval was therefore not required. Data was analysed using R version 4.1.3 [29]. 

## Results

### Population demographics

**Fig. 1:**
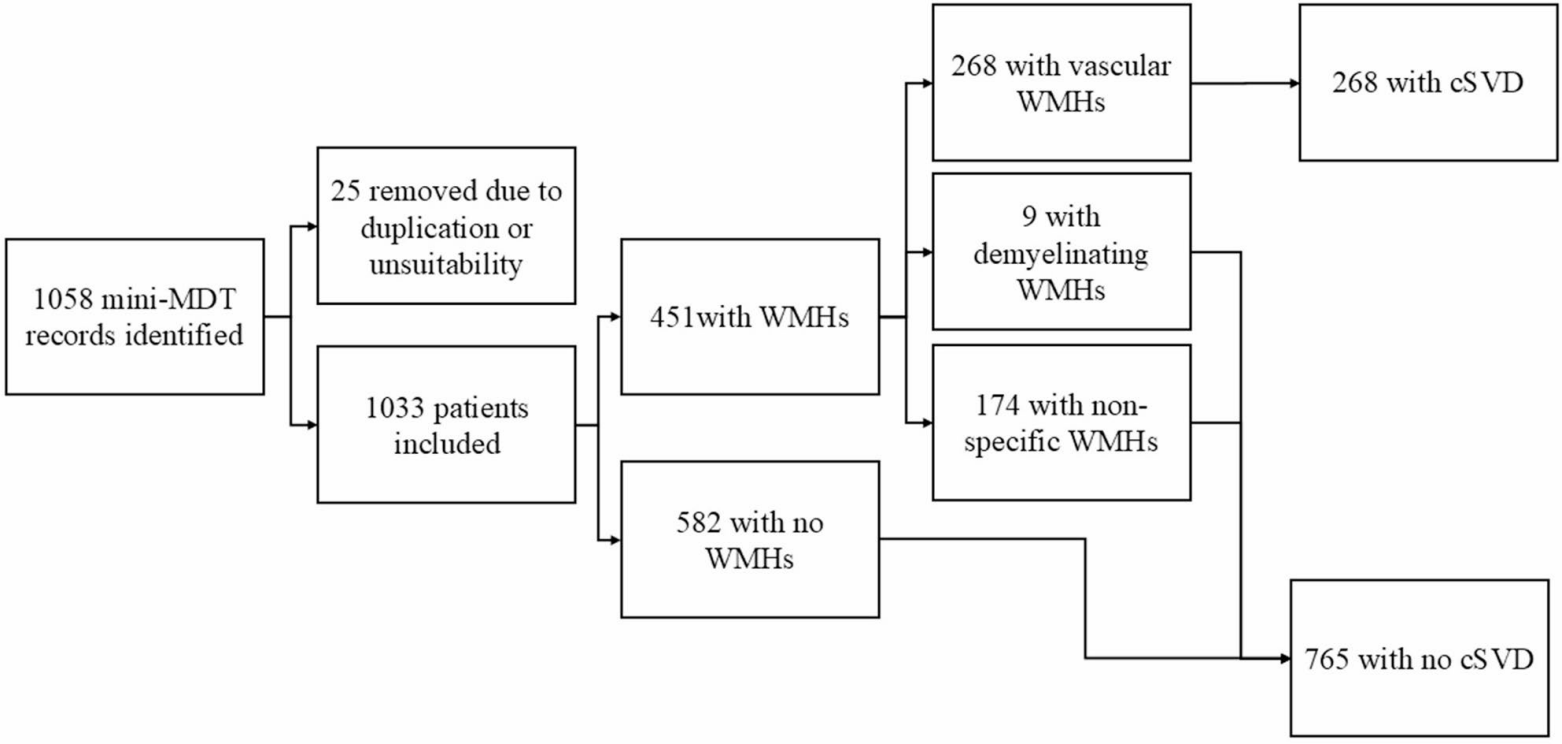
Study flow chart

We retrospectively identified a total of 1058 mini-MDT records from this 30-month period (see Fig. 1). We excluded 21 records which were incomplete (ensuring there was no missing data for any variables) or duplicated and 4 due to subjects having undergone a CT brain scan in view of severe claustrophobia, contraindications to MRI (e.g. permanent pacemaker) or patient preference. Baseline demographics and referral criteria are shown in Table 2.

**Table 2:** Patient demographics and referral criteria

**Demographics**			
	**Mean (SD)/** *n* **(%)**		
Age	51.3 (18.3)		
Age group			
Under 50	472 (46%)		
50–59	185 (18%)		
60–69	186 (18%)		
70–79	132 (13%)		
80 and above	58 (6%)		
Sex			
Male	362 (35%)		
Female	671 (65%)		
WHO Performance score (see Table 1)			
0	709 (81%)		
1	128 (15%)		
2	32 (4%)		
3	9 (1%)		
4	0 (0%)		
**Deprivation decile**	**Index of Multiple Deprivation (IMD)**	**Health Deprivation and Disability**	**Education, Skills, and Training Deprivation**
1 (Most deprived)	83 (8%)	128 (12%)	49 (5%)
2	102 (10%)	129 (12%)	62 (6%)
3	95 (9%)	120 (12%)	116 (11%)
4	71 (7%)	110 (11%)	79 (8%)
5	62 (6%)	104 (10%)	45 (4%)
6	49 (5%)	181 (18%)	53 (5%)
7	100 (10%)	147 (14%)	137 (13%)
8	153 (15%)	93 (9%)	137 (13%)
9	159 (15%)	21 (2%)	168 (16%)
10 (Least deprived)	159 (15%)	0 (0%)	187 (18%)
**Referral Criteria**	**Number (%)**		
Recent headache	662 (64%)		
Posture related headache	333 (32%)		
Progressive neurological deficit	238 (23%)		
Cognitive impairment	224 (22%)		
Vomiting	138 (13%)		
Drowsiness	104 (10%)		
Pulsatile tinnitus	58 (6%)		
Personality changes	52 (5%)		
Seizures	41 (4%)		
Cranial nerve palsy	23 (2%)		
Unilateral deafness	21 (2%)		
Papilloedema	10 (1%)		

As expected, many proformas had multiple 2WW referral criteria selected. Headache and symptoms raising suspicion of raised intracranial pressure represented the most frequent concern (headaches of recent onset *n* = 662, posture related headache *n* = 333, vomiting *n* = 138, pulsatile tinnitus *n* = 58 and papilloedema = 10).

### Prevalence of WMHs and cSVD

**Fig. 2:**
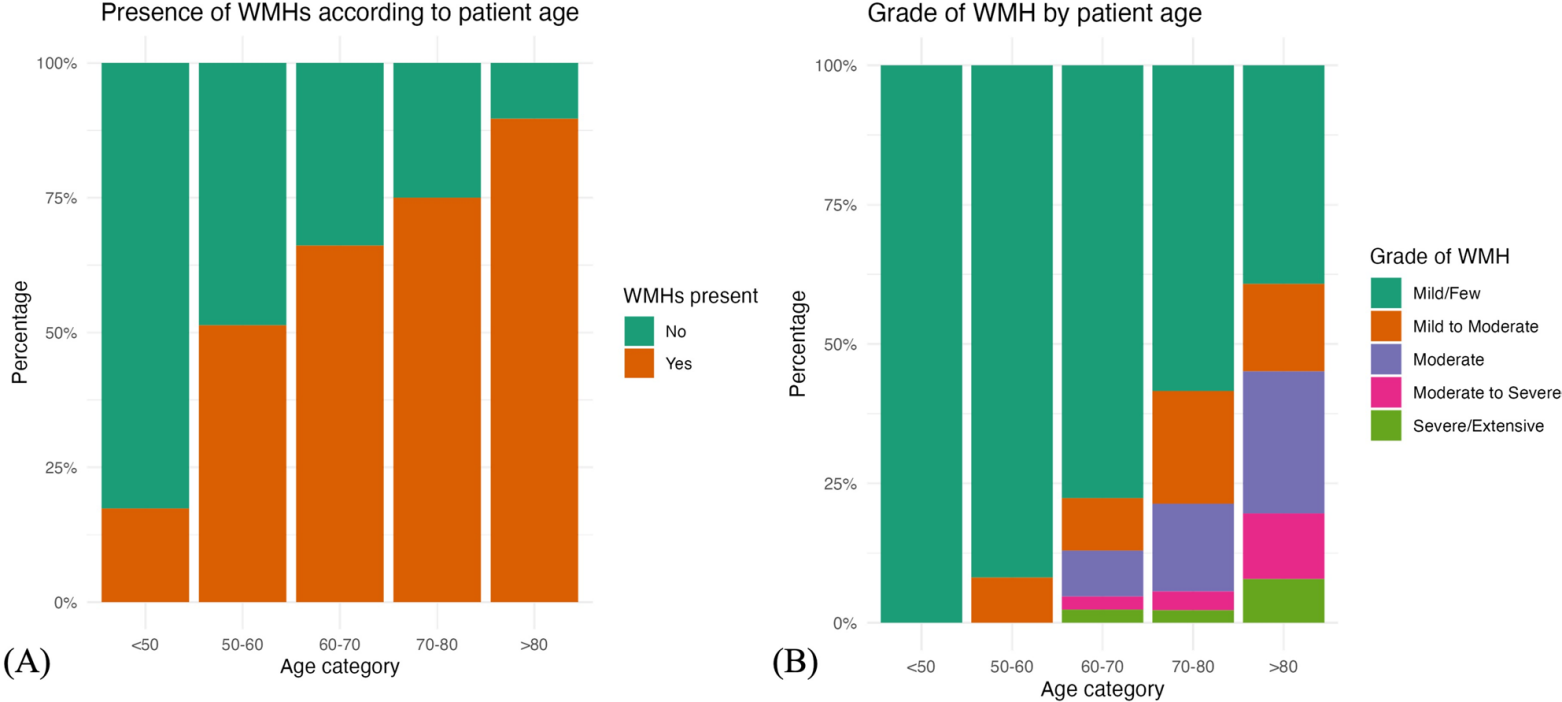
The prevalence (**A**) and grade (**B**) of WMHs according to patient age group

Overall, 451 (43.7%) scans demonstrated evidence of WMHs with 59% of these scans deemed in keeping with cSVD (see Table 3). The prevalence of WMHs increased with age, from approximately 20% of patients under 50 years old to almost 90% of those over 80 years (see Fig. 2a). Advancing age also appeared to correlate with increasing radiological burden of WMHs (see Fig. 2b).

**Table 3:** Population demographics and prevalence of WMH by age group and radiological aetiology

	Overall	Males	Females
WMHs by age group			
<50 (%)	82/472 (17.4)	24/142 (16.9)	58/330 (17.6)
50–59 (%)	95/185 (51.4)	36/71 (50.7)	59/114 (51.8)
60–69 (%)	123/186 (66.1)	46/69 (66.7)	77/117 (65.8)
70–79 (%)	99/132 (75.0)	35/55 (63.7)	64/77 (83.1)
>80 (%)	52/58 (89.7)	23/25 (92.0)	29/33 (87.9)
WMHs by aetiology			
Non specific (%)	174/451 (38.6)	63/164 (38.4)	111/287 (38.7)
Demyelinating (%)	9/451 (2.0)	4/164 (2.4)	5/287 (1.7)
cSVD (%)	268/451 (59.4)	97/164 (59.1)	171/287 (59.6)
WMHs by age group and aetiology			
	Non specific	cSVD	Demyelinating
<50 (%)	71/82 (86.6)	6/82 (7.3)	5/82 (6.1)
50–59 (%)	56/95 (59.0)	37/95 (38.9)	2/95 (2.1)
60–69 (%)	36/123 (29.3)	85/123 (69.1)	2/123 (1.6)
70–79 (%)	10/99 (10.1)	89/99 (89.9)	0/99 (0)
>80 (%)	1/52 (1.9)	51/52 (98.1)	0/52 (0)

**Fig. 3:**
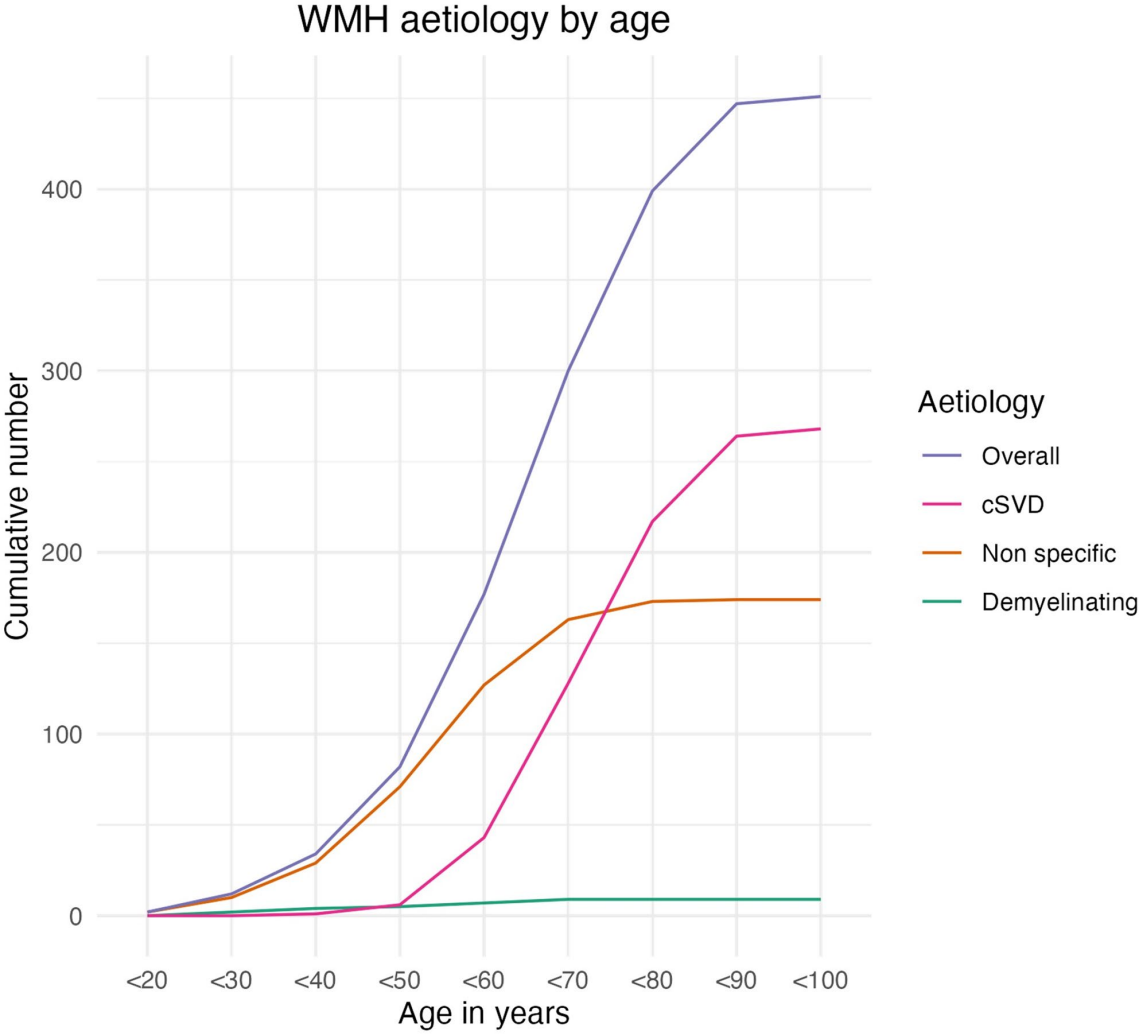
The cumulative radiological WMH aetiology according to age

Age also appeared to influence the radiological aetiology of WMHs (see Fig. 3). Under the age of 50, WMHs were considered non-specific in 86% of scans where they were present, compared to < 30% in those over 50. Indeed after age 50, the majority of WMHs were attributed to cSVD, rising to > 90% of cases in patients over 70 years old.

Chi squared results demonstrated no significant difference in the presence of overall WMHs (*p* = 0.47) between males (45%) and females (43%). Similarly, there was no significant difference identified in the presence of cSVD (*p* = 0.70) between males (27%) and females (25%).

**Fig. 4:**
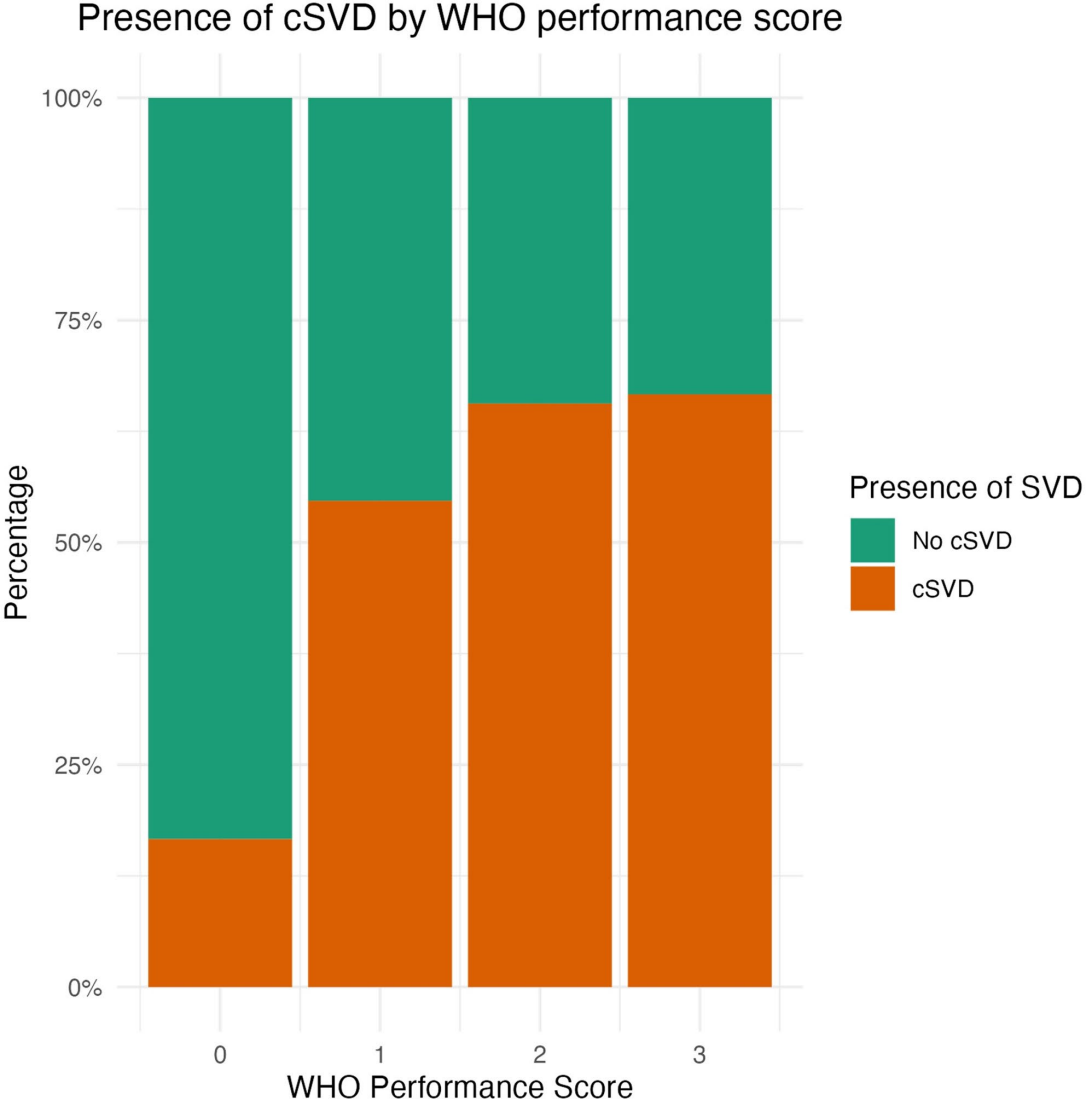
Presence of cSVD by WHO performance score

The proportion of patients with cSVD appeared higher in participants with WHO performance score > = 1 with 57% having cSVD compared to 17% with a score of 0 (see Fig. 4), indicating a degree of restricted activities, this is explored further in the context of other variables in the regression analysis.

### Risk factors for cSVD

We performed multivariable logistic regression modelling to investigate which patient demographic factors impacted the likelihood of identifying WMH with a cSVD based aetiology; see table A in the supplementary materials. We found a significant relationship between age and presence of cSVD with a 14.5% increase in the odds ratio with each increased year of age (*p* < 0.001). When we performed a sensitivity analysis whereby 10% of the radiological cSVD diagnoses were randomly inverted, age remained significant (*p* < 0.001). Higher EST decile (indicative of higher levels of deprivation in terms of education, skills and training) was also associated with an increased risk of cSVD (*p* < 0.05). Conversely, higher IMD decile was associated with a reduced risk of cSVD (*p* < 0.05). HDD decile was non-significant (*p* = 0.36). Patient sex (*p* = 0.07) and WHO performance score (*p* = 0.55) also did not reach statistical significance. Considering the magnitude of impact age had upon cSVD, we analysed a model to only account for this factor. Whilst age remained a significant factor (*p* < 0.001), inclusion of additional demographic factors improved model fit, using AIC as a metric to avoid overfitting.

We also performed multivariable logistic regression modelling conditional on age to identify whether any of the referral criteria were associated with WMH with an cSVD based aetiology. This revealed ‘headaches of recent onset’ (*p* = 0.023) as negatively associated with reduced cSVD prevalence. (Supplementary Table B).

Whilst the structured referral proforma does include designated fields for the recording of vascular risk factors and previous stroke, these were inconsistently documented and therefore excluded from our analysis.

### Additional imaging findings

In addition to WMHs, radiological abnormalities were identified on 438 (42.4%) scans (see Supplementary Table C). These were most commonly either ear, nose and throat abnormalities (e.g. sinus thickening), soft radiological signs of raised intracranial pressure (e.g. partially empty sella turcica, optic nerve tortuosity, enlarged optic nerve sheath or flattening of the optic nerve head) or vascular abnormalities (e.g. aneurysm, arteriovenous malformations or venous anomalies). Approximately 3% of scans demonstrated neoplastic disease (2.5% primary/0.5% secondary brain tumours). It should be borne in mind that only 1.3% of scans demonstrated suspected malignant brain tumour or metastases. Forty-five (4.4%) scans revealed > 1 radiological abnormality.

## Discussion

This study was a service evaluation based on a prospectively collected dataset from routine clinical practice. Using routinely collected data we demonstrate a high prevalence of WMHs and cSVD in the elderly population, comparable to prospective cohort studies and the wider literature [24]. We also emphasise that WMHs are prevalent amongst the younger population.

In younger patients, WMHs are frequently deemed non-specific. Based on our findings, it is conceivable that many of these scans are actually indicative of mild cSVD. This may highlight a source of bias evident in routine neuroradiology reporting with reluctance to label changes as suggestive of cSVD in younger age categories combined with the limitations of current imaging techniques. These patients may benefit from simple, targeted interventions to mitigate vascular risk such as screening for hypertension, smoking cessation and advocating regular exercise. Similarly, whilst rigorous blood pressure targets may not be suitable for all elderly patients who may be prone to postural hypotension and falls, this may be more suitable in this lower age group.

Communicating the importance of low burden covert cSVD to seemingly asymptomatic patients can be challenging. However, WMHs on conventional imaging may reflect only the ‘tip of the iceberg’ when compared to the wider microstructural changes within normal appearing white matter [30, 31]. Given the implications for subsequent stroke, dementia, neurodegeneration, physical dependency and death, earlier identification and discussion may serve as an opportunity for proactive intervention. Similarly, increased identification would facilitate recruitment to future cSVD research such as investigating genetic components, longitudinal WMH changes, novel imaging techniques to serve as surrogate trial end-points and targeted therapeutics [12, 32, 33]. 

We also found that cSVD is associated with performance score, indicating a degree of physical dependency or frailty in this group of patients. Further research is required to fully understand this finding, which may simply represent the typical ‘cSVD syndrome’. However, this does serves as a reminder of the potential benefits of preventing cSVD for preserving quality of life on a patient level and reducing the overall care burden on a population scale.

The association between LSOA-linked deciles of deprivation and cSVD was less clear, with increasing levels of health, skills and training associated with higher levels of cSVD with the converse true for IMD decile. Although interesting as a proof of concept, we acknowledge these findings may be artefact; a product of the relatively small geographical area covered by the local 2WW pathway and visualised by the vague correlations seen in Fig. 4.

Similarly, the associations between cSVD and specific referral criteria should be interpreted with caution. Notwithstanding the often pragmatic nature of referrals generated by routine clinical practice and the influence of age, this reflects the typical profile of patients referred via the 2WW CNS pathway with headache and the local referral architecture (e.g. suspected seizures are instead directed via the ‘first fit’ clinic). Nevertheless, larger studies across multiple referral streams and centres may improve this analysis. Developments in the organisation of routinely collected data within healthcare systems should facilitate such analyses.

Given the deliberately selective nature of 2WW CNS referrals, we expected an increased number of additional imaging findings when compared with asymptomatic, prospective population studies [34]. The 3% rate of neoplastic disease is in line with suspected cancer referral criteria and comparable to a previous local study of this referral stream [27]. Nevertheless, we would argue that patients referred on this pathway (i.e. patients referred for imaging after seeking medical attention on the basis of being symptomatic) are representative of routine practice. With an increasing number of MRI scans performed each year, our findings vis a vis prevalence and nature of imaging findings may assist with counselling patients regarding the risk of incidental findings.

We acknowledge that this study does have limitations. Primarily, these are due to the variable granularity of routinely collected data, the single geographical area represented by referrals, the notional pre-requisite for neurological symptoms suspicious for an underlying CNS malignancy and the unblinded analysis of imaging using a visual rating scale. We acknowledge that the use of routinely collected data can be seen as a limitation and may impact the reproducibility of our results, for example with the absence of volumetric analysis of WMHs, but a key strength of our approach is that it helps to inform the potential implications of findings as described in routine clinical reports. Future analysis may benefit from a standardised approach to considering aetiology and quantifying WMHs lesions such as the Fazekas score [35], with consideration of wider imaging markers of cSVD, and commenting on their distribution. We lacked detailed data on comorbidities, medication use and lifestyle factors, which can influence WMH prevalence and characteristics. However, despite these factors we returned similar values to prospective studies with statistically significant outcomes.

The surprising prevalence of WMHs in younger patients underlines the need to ensure careful clinical characterisation and further research to ensure that potential opportunities for intervention are not missed. While WMHs in the younger population are often deemed non-specific, our findings are in keeping with at least a proportion being due to cSVD, so it is likely enacting simple, standardised lifestyle interventions, and exclusion of hypertension, are reasonable in order to mitigate vascular risk. However, as a retrospective analysis, we acknowledge the inherent limitation in establishing causal relationships between WMHs and cSVD, which necessitates cautious interpretation of our findings.

This study signals the potential for routinely collected data to further our understanding of cSVD prevalence, demographics, risk factors and clinical syndromes alongside facilitating targeted recruitment of well-phenotyped cSVD patients to future research studies.

## Supplementary Information

Supplementary Material 1.

## Data Availability

No datasets were generated or analysed during the current study.
