# Supplementary material for: Prevalence of white matter hyperintensities and radiological cerebral small vessel disease: an insight from routinely collected data
Source: BMC Neurol. 2025 Dec 19;26:47. doi: 10.1186/s12883-025-04557-y (PMC12831340; doi:10.1186/s12883-025-04557-y)
Supplement: Supplementary file 1 — Supplementary Material 1. [file 12883_2025_4557_MOESM1_ESM.docx]

| **Characteristic** | **OR***^1^* | **95% CI***^1^* | **p-value** |
| --- | --- | --- | --- |
| Age | 1.15 | 1.12, 1.17 | <0.001 |
| Sex |  |  |  |
| Female | — | — |  |
| Male | 0.67 | 0.43, 1.04 | 0.075 |
| WHO Performance Score | 1.12 | 0.78, 1.61 | 0.6 |
| Index of Multiple Deprivation Decile | 0.79 | 0.62, 1.00 | 0.050 |
| Education, Skills, and Training Deprivation Decile | 1.21 | 1.02, 1.44 | 0.032 |
| Health Deprivation and Disability Decile | 1.10 | 0.89, 1.36 | 0.4 |
| *^1^*OR = Odds Ratio, CI = Confidence Interval | | | |

***Supplementary Table A****: Model 1 – Patient characteristics*

| **Characteristic** | **OR***^1^* | **95% CI***^1^* | **p-value** |
| --- | --- | --- | --- |
| Progressive neurological deficit | 0.98 | 0.53, 1.81 | >0.9 |
| Recent headaches | 0.52 | 0.30, 0.91 | 0.023 |
| Vomiting | 1.52 | 0.77, 3.01 | 0.2 |
| Drowsiness | 0.44 | 0.19, 1.02 | 0.057 |
| Posture related headache | 1.04 | 0.63, 1.70 | 0.9 |
| Pulsatile tinnitus | 1.58 | 0.67, 3.70 | 0.3 |
| Papilloedema | 0.44 | 0.01, 14.3 | 0.6 |
| Unilateral deafness | 0.55 | 0.13, 2.33 | 0.4 |
| Personality changes | 0.89 | 0.36, 2.19 | 0.8 |
| Seizures | 0.76 | 0.24, 2.37 | 0.6 |
| Cognitive impairment | 0.68 | 0.39, 1.19 | 0.2 |
| Cranial nerve palsy | 1.47 | 0.38, 5.63 | 0.6 |
| *^1^*OR = Odds Ratio, CI = Confidence Interval | | | |

***Supplementary Table B:*** *Model 2 – Symptoms (all Odds Ratios are relevant to not having the symptom)*

| **Additional findings:** | **N (% of 1033 scans)** |
| --- | --- |
| Recent infarct or haemorrhage | 7 (0.7) |
| Neoplasm: |  |
| Primary brain tumour | 26 (2.5) |
| - Of which are meningioma | 18 (1.7) |
| Metastatic brain tumour | 5 (0.5) |
| Cerebellar descent / tonsillar ectopia / Chiari malformation | 38 (3.7) |
| ENT abnormality (e.g. sinus thickening) | 81 (7.8) |
| Developmental brain anomaly | 9 (0.9) |
| Pituitary signal change / suspected adenoma | 19 (1.8) |
| Soft radiological signs of raised intracranial pressure | 89 (8.6) |
| Microhaemorrhages or suggestive of cerebral amyloid angiopathy | 11 (1.1) |
| Radiological signs of normal pressure hydrocephalus | 7 (0.7) |
| Non-specific gliosis | 44 (4.3) |
| Arachnoid cyst | 11 (1.1) |
| Pineal cyst | 18 (1.7) |
| Aneurysm / venous anomaly / AVM | 46 (4.5) |
| Bone lesion | 13 (1.3) |

***Supplementary Table C:*** *Additional radiological findings on routine 2WW brain imaging (soft signs of raised intracranial pressure suggestive of conditions such as idiopathic intracranial hypertension include a partially empty sella turcica, optic nerve tortuosity, enlarged optic nerve sheath and flattening of the optic nerve head)*
